# Improving perception and confidence towards bystander cardiopulmonary resuscitation and public access automated external defibrillator program: how does training program help?

**DOI:** 10.1186/s12245-020-00271-3

**Published:** 2020-03-17

**Authors:** Siew Yee Liaw, Keng Sheng Chew, Ahmad Zulkarnain, Shirly Siew Ling Wong, Nariman Singmamae, Dev Nath Kaushal, Hiang Chuan Chan

**Affiliations:** 1 Emergency Medicine and Trauma Department, Sarawak General Hospital, Jalan Hospital, Sarawak, 93586 Malaysia; 2 Faculty of Medicine, Universiti of Malaya, Jalan Universiti, Kuala Lumpur, 50603 Malaysia; 3 Faculty of Medicine and Health Sciences, Universiti Malaysia Sarawak, Jalan Datuk Mohammad Musa, 94300 Kota Samarahan, Sarawak Malaysia; 4 Faculty of Economics & Business, Universiti Malaysia Sarawak, Jalan Datuk Mohammad Musa, Kota Samarahan, 94300 Sarawak Malaysia

**Keywords:** Automated external defibrillator, Cardiopulmonary resuscitation, Theory of planned behavior, Bystander, Willingness

## Abstract

**Background:**

In conjunction with an automated external defibrillator (AED) placement program at various locations within a public university in Malaysia, a series of structured training programs were conducted. The objectives of this study is to (1) evaluate the effectiveness of a structured training program in improving the perception of the importance of AED and cardiopulmonary resuscitation (CPR), (2) evaluate the confidence of the employees in using an AED and performing bystander CPR, (3) identify the fears and concerns of these employees in using AED and performing CPR, and (4) determine the perception of these employees towards the strategy of the AEDs placed at various locations within the university.

**Methods:**

In this single-center observational study, a validated questionnaire aimed to assess the university employees’ attitude and confidence in handling AED and performing CPR before (pre-test) and immediately after (post-test) the training program was conducted.

**Results:**

A total of 184 participants participated in this study. Using the Wilcoxon signed-rank test, the training programs appeared to have improved the perception that “using AED is important for unresponsive victims” (*z* = 4.32, *p* < 0.001) and that “AED practice drills should be performed on a regular basis” (*z* = − 2.41, *p* = 0.02) as well as increased the confidence to perform CPR (*z* = − 8.56, *p* < 0.001), use AED (*z* = − 8.93, *p* < 0.001), identify victims with no signs of life (*z* = − 7.88, *p* < 0.001), and the willingness to perform CPR and AED without hesitancy (*z* = − 8.91, *p* < 0.001). Fears and concerns on performing CPR and using AED also appeared to have been significantly reduced, and the perception on placement strategies of these AEDs was generally positive.

**Conclusion:**

Using the theory of planned behavior as the explanatory framework, training programs appear to be helpful in improving the perception and the confidence of the participants towards performing CPR and using AED through the promotion of positive attitude, positive societal expectation, and a positive sense of empowerment. But whether this positive effect will translate into actual CPR performance and AED application in a real cardiac arrest is yet to be seen.

## Background

As ventricular fibrillation (VF) remains, the commonest initial rhythm in sudden out-of-hospital cardiac arrest (OHCA) [1, 2], cardiopulmonary resuscitation (CPR), and defibrillation are two pivotal life-saving interventions that increase the chance of survival to hospital discharge [3]. To be effective, these two interventions have to be delivered within minutes from the onset of cardiac arrest [1, 4] as it has been shown that with every passing minute without the initiation of cardiopulmonary resuscitation and defibrillation, the chance of survival declines by about 7–10% [2].

Hence, in order to minimize the time delay from collapse to CPR and defibrillation, these two interventions should be promptly initiated by bystanders [1, 5]. For years, the American Heart Association (AHA) recommends for AEDs to be placed in areas with a high risk of cardiac arrest [2, 6–9]. These AEDs should be easily locatable, placed within the vicinity of the victim, and can be accessible by the bystanders at all times [1, 5, 10–12].

Unfortunately, despite the widespread dissemination of AEDs, the effective use of these AEDs can still be limited if they are placed inappropriately [7–9, 13–17]. In other words, the preparedness of bystander to use AEDs is as important as the placement of the AEDs itself. With proper training and education, bystanders can use these AEDs safely and effectively [18, 19].

Kronick et al. outlined four essential components for a successful implementation of a public access AED program: (1) developing a practice plan to respond to an cardiac arrest; (2) training of identified personnel such as the security guards, police, and administrative employees as lay rescuers in the skills of CPR and the use of the AED; (3) establishing an integrated link with the local EMS system; and (4) ensuring a program of ongoing quality improvement [20].

In Malaysia, the implementation of public access AED is still a relatively new endeavor. Realizing the importance of public access AED, Universiti Malaysia Sarawak (UNIMAS), a public university in the state of Sarawak, Malaysia, recently implemented the public access AED program and placed AEDs at various strategic locations around the campus [21]. To ensure that non-healthcare employees of UNIMAS are adequately equipped to respond to a sudden OHCA victim, a series of structured training programs were conducted for employees from different faculties and departments in UNIMAS.

Each training program consists of a 60-min classroom lecture highlighting on the importance of bystander CPR and prompt delivery of shock using AED. The contents of this lecture are based on compiled AHA teaching materials, delivered through slides and video presentations. This was followed by a hands-on session consisting of skill training on CPR and AED application on manikins.

We embarked on this study with the objectives of (1) evaluating the effectiveness of structured training program in improving the perception of the importance of AED and CPR, (2) improving the confidence of the employees in using an AED and performing CPR, (3) identifying the fears and concerns of these employees in using AED and performing CPR, and (4) determining the perception of these employees towards the placement strategies of the AEDs placed at various locations within the university.

## Methods

This was a single-center observational study conducted from February 2018 to November 2018 in conjunction with the CPR and AED skills training program conducted for employees of UNIMAS. Prior to the commencement of the training, the questionnaire forms were distributed to the participants. The participants filled in the questionnaire forms before (pre-test) and after (post-test) the training program. Ethical approval was obtained from the Medical Research and Ethics Committee, Ministry Of Health Malaysia, and the study was registered under the Malaysian National Medical Research Register (NMRR, website URL: www.nmrr.gov.my) with the research number of NMRR-16-696-39041.

### Participants

Employees from UNIMAS who participated in the training program on how to use the AED and perform CPR were also invited to participate in this study. Healthcare employees like doctors and paramedic staff were excluded from the study. Informed consent was obtained from all participants prior to their participation.

The sample size for this study was estimated to be 158 based on a previous study on the rate of bystander CPR (of approximately 9%) in a community in Malaysia [22]. Single proportion formula with a 95% confidence interval, margin error of 0.05, and a dropout rate of 20% was applied. A convenient sampling method was adopted as all participants in this study were also participants of the CPR and AED skills training program.

### Materials

The validated questionnaire used in this study consists of 2 parts. The first part of the questionnaire is to gauge the perceptions of the participants on the implementation strategies of the AEDs placed in UNIMAS campus. The second part of the questionnaire is to assess participants’ attitude and confidence in handling AED and performing CPR before (pre-test) and after (post-test) the training program as well as their fears and concerns in using AED and performing CPR.

### Procedure

The self-administered questionnaire survey forms were distributed by one of the researchers (SYL) in conjunction with the CPR and AED skills training programs. Prior to commencing this training program, the participants were asked to complete the pre-test section on the perception, confidence, and fears and concerns on performing CPR and using AED. The post-test section was filled immediately after the completion of the training program. Informed consent was obtained from all participants. Participants were informed that his or her data would be used anonymously for subsequent analysis, presentation, and publication.

## Results

A total of 184 administrative employees from UNIMAS participated in this study. The mean age group of the participants was 37.6 years (SD ±  6.85), and 100 out of 184 of them (54.3%) were male participants. The level of education ranged from secondary school level to postgraduate level, with a majority of them (68 or 37%) being diploma holders. With regard to their working experience in UNIMAS, the mean working experience was 11.5 years (SD ± 5.43).

Non-parametric tests were used as the data was not normally distributed. Using the Wilcoxon signed-rank test to determine the effectiveness of the training program to improve the perception of using AED and performing CPR (“pre-test” vs “post-test”), statistically significant improvement was noted in the perception that “using an AED is important for any unresponsive victims” and that “AED practice drills should be performed on a regular basis”, *z* = − 4.32, *p* < 0.001 and *z* = − 2.41, *p* = 0.02, respectively. Similarly, with regards to confidence in using the AED and performing CPR, our results show that the training program improved the confidence to perform CPR (*z* = − 8.56, *p* < 0.001), confidence to use AED (*z* = − 8.93, *p* < 0.001), confidence to identify signs of victims with no signs of life (*z* = − 7.88, *p* < 0.001), and willingness to perform CPR and AED without hesitancy (*z* = − 8.91, *p* < 0.001). The details of the all pre- vs post-training analysis using the Wilcoxon signed-rank test are tabulated in Table 1. Similarly, using the Wilcoxon signed-rank test, the training program also seems to have significantly reduced the degree of fear and concerns in performing CPR and using AED (refer to Table 2). With regards to the placement strategies of these AED, more than 50% of the participants agreed that the AEDs were visibly placed (125 out of or 67.9%) in a secure location (122 participants or 66.3%) with clear signage (113 participants or 61.4%), good accessibility (112 participants or 60.7%), and easy-to-follow instructional poster (99 participants or 53.8%) (see Table 3).

**Table 1:** Effectiveness of training to improve the perception and confidence in using AED and performing bystander CPR

Variable	Number of cases where post-training score > pre-training score (mean rank)	Number of cases where post-training score < pre-training score (mean rank)	***z*** value	***p*** value
**Perception of the participants**				
AED is lifesaving	30 (25)	19 (25)	1.57	0.12
It is important for an AED to be available in the place where I work	26 (21)	15 (21)	1.72	0.09
Using an AED is important for any unresponsive victims	42 (27.4)	11 (25.5)	4.32	< 0.001
Person who handles an AED requires formal training	29 (23.8)	18 (24.3)	1.508	0.31
AED practice drills should be performed on a regular basis	33 (26.0)	17 (24.5)	2.41	0.02
**Confidence of the participants**				
I am confident to perform CPR	97 (50.4)	3 (52.7)	8.56	< 0.001
I am confident to use an AED	107 (56.1)	4 (53.0)	8.93	< 0.001
I am confident in recognizing victim with no signs of life	93 (52.5)	(41.1)	7.88	< 0.001
I will not hesitate to perform CPR and use AED on an unresponsive victim	106 (55.6)	4 (53.5)	8.91	< 0.001

Note: the higher the score, the positive is the response

**Table 2:** Fears and concerns of participants in performing CPR and using AED

Variable	Number of cases where post-training score > pre-training score (mean rank)	Number of cases where post-training score < pre-training score (mean rank)	***z*** value	***p*** value
**Fears and concerns**				
Infection from performing CPR	24	63	− 4.342	< 0.001
Injuring victim due to performing CPR	8	100	− 8.502	< 0.001
Injuring own self due to performing CPR	15	63	− 5.380	< 0.001
Injuring victim due to using AED	9	100	− 8.451	< 0.001
Injuring own self due to using AED	11	75	− 6.642	< 0.001
Getting sued due to performing CPR	8	108	− 8.847	< 0.001
Getting sued due to using AED	9	107	− 8.517	< 0.001

Note: the higher the score, the more concerned the participants

**Table 3:** Participants’ opinions on the AED placement strategies

Domain	Criteria	Number of participants who agreed (%)
Visibility	The AED is placed in a clearly visible location	125 (67.9%)
Signage	The signage that shows the location of the AED is clear	113 (61.4%)
Accessibility	The AED is located in a location that is easily accessible at all times (including after office hours)	112 (60.7%)
Instruction	The steps in the AED instructional poster on how to use the AED are easy to follow	99 (53.8%)
Security	The AED is located at a secure area	122 (66.3%)

Using Spearman’s rank-order correlation, the correlation between the confidence to perform CPR with the confidence to use AED was strong, *r*_*s*_(182) = 0.87, *p* < 0.001 as well as the correlation between the confidence to perform CPR with the confidence to recognize victim with no signs of life, *r*_*s*_(182) = 0.65, *p* < 0.001. Similarly, the correlation between the confidence to use AED with the willingness to initiate the use of AED without hesitancy was strong with *r*_*s*_(182) = 0.80, *p* < 0.001.

## Discussion

To answer the question of how a structured training program can help to improve the willingness of the participants to perform CPR and to use public access AEDs, two theoretical frameworks were adopted, i.e., Kirkpatrick’s training evaluation model [23] and the theory of planned behavior (TPB) [24]. Kirkpatrick’s training evaluation model is a tool used to evaluate the levels of participants’ responses towards a training program [23]. In this model, level 1 is known as the “reaction” level. This level is merely an experiential level (i.e., whether the participants have had good experience and engagement in the training activities). Real learning might or might not have taken place. Level 2 is known as the “learning level.” At this level, participants should have, to a large degree, acquired the intended knowledge and skills outlined in the training objectives as well as have instilled some degree of positive attitude, confidence, and willingness of the participants to act on the desired learning goals. Level 3 (“behavior level”) measures the degree to which participants can actually apply what they had learned during the training (i.e., actually performing CPR and using public access AED in a real OHCA). Level 4 is known as the “results level.” This level essentially measures the positive impact of the training for the community as a result from the acquired knowledge and skills (i.e., the actual increase in OHCA survival rate). In this regard, we believed that at this current moment, our training program had achieved at least level 2 of Kirkpatrick’s training evaluation model as participants had demonstrated improved scores in their knowledge, skills, and attitude after the training (compared to before the training).

The theory of planned behavior (TPB) is a three-dimensional theoretical framework developed by Ajzen to understand and predict human behavior [24]. According to Ajzen, the immediate precursor that determines whether a person would decide to act on a particular volitional behavior (i.e., in this regard, the act of performing CPR and applying AED when an actual cardiac arrest occurs) is known as the “intention” to perform that behavior [24]. The intention of this study refers to the willingness to perform CPR and AED without hesitancy. In this regard, the training programs did indeed seem to be effective in promoting the positive intention of the willingness to perform CPR and use AED as shown in the pre- vs post-training analysis in Table 1. This intention (willingness to perform CPR and using AED), according to the TPB, in turn, is said to be influenced by three determinants: (1) attitude, (2) subjective norm, and (3) perceived behavioral control.

A person’s attitude towards a particular behavior (i.e., performing CPR and applying AED when an actual cardiac arrest occurs) is developed, either favorably or unfavorably, from the beliefs that he or she holds towards that behavior as well as other attributes associated with the behavior (e.g., the legality concerns and fears of injuring the victims through CPR and AED use as shown in Table 2, which may negatively impact one’s attitude towards this behavior). Behaviors that are believed to have desirable outcomes (i.e., promptly performing CPR and using AED can save lives) tend to be favored while behaviors that are believed to have undesirable outcomes are not favored [24]. Hence, it is imperative to instill the notion that prompt initiation of bystander CPR and the prompt use of AED are of paramount importance.

As human is by nature a social creature who highly regards what society and his or her significant people around him or her (e.g., family, friends, and colleagues) think of his or her behavior (i.e., the social normative), it is essential that the element of social interaction (such as the group practice of CPR and AED together) is incorporated into the training in order to help dispel the oddity of performing bystander CPR and using AED in public. This is because, according to TPB, if society expects or favors a particular behavior (in this case, the societal norm or expectation to promptly initiate bystander CPR and apply AED), then it is more likely that the individual will have the positive intention to act on this behavior should the need arises [24]. Hence, while we did not explicitly measure the influence of social interaction on the willingness to perform bystander CPR or using AED, we believe that the social interaction and group practice in our training program are important to promote the positive intention of willingness to perform CPR and using AED.

The perceived behavioral control, on the other hand, refers to the perceived empowerment or ability to actually perform or engage in the behavior (i.e., performing bystander CPR and using AED) based on the resources and opportunities available to him or her at that particular time [24]. A bystander who felt empowered or felt that he had the capability to execute a particular behavior is more likely to perform it when the need arises. In this regard, it is imperative to ensure that the AEDs are placed strategically (refer to criteria in Table 3) and the employees are given sufficient hands-on training to open the AED cover, to touch and to push the specific buttons of the AEDs located within their reach. This will give them a sense of control or empowerment as they would be familiar with the location as well as the specific AED placed in that location. Furthermore, according to Ajzen, perceived behavioral control is also very much dependent on the perceived probability of succeeding in executing a given task [24]. As such, it is also important to inculcate the confidence of these employees to perform CPR and to use the AED. In this regard, our training program did indeed seem to improve the confidence of the employees to use the AED (Table 1). In fact, positive correlation was demonstrated between the confidence to perform CPR with the confidence to use AED, the correlation between the confidence to perform CPR with the confidence to recognize victim with no signs of life, and the correlation between the confidence to use AED with the willingness to initiate the use of AED without hesitancy suggesting the importance of seeing these 3 skills as a three-legged stool that should be developed in tandem.

**Fig. 1:**
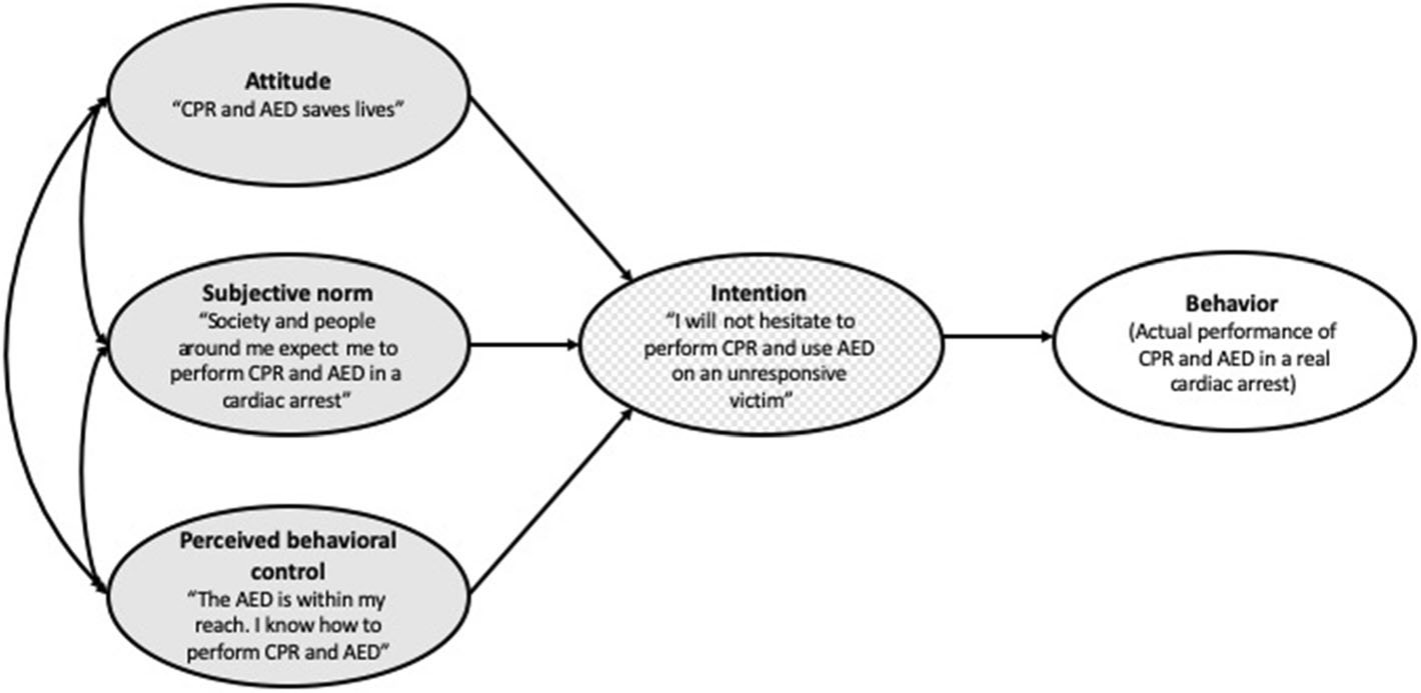
Theory of planned behavior as a framework to explain how a training program can have a positive impact on the intention to perform CPR and use AED

In summary, to be effective in improving the perception and confidence of the public towards bystander CPR and using AED, the following elements should be incorporated in the training: (1) instilling positive attitude and perception on CPR and AED based on latest scientific evidences that CPR and AED save lives as well as allaying fears and concerns related to CPR and AED, (2) promoting positive societal expectation that prompt bystander CPR and AED is called for when someone collapses, and (3) promoting a sense of empowerment and confidence that using AED located is within their reach and capability (see Fig. 1 on how TPB can be used as an explanatory framework to build a training program).

A number of limitations deserve mentioning. First, this study involved participants from only one center and merely involved employees working in an academic center. Hence, it might not be generalizable to include perception from the other communities and from participants of different levels of educational background. Furthermore, the training program could only possibly achieve up to the “intention” level in the TPB framework and up to “level 2—learning” in the Kirkpatrick training evaluation model. This suggests that at this current moment, the effectiveness of the training program in increasing the actual performance of CPR and AED when an actual OHCA occurs is yet to be known. As stated by Ajzen, for accurate behavioral prediction (on the likelihood to perform CPR and use AED should an actual cardiac arrest occurs), the intentions (i.e., the willingness to perform CPR and use AED) and perceived behavioral control (i.e., confidence to perform CPR and use AED) should remain stable in the interval between the assessment of these intentions and perceived behavioral control until the actual performance of an intended behavior [24]. Various intervening events (e.g., seeing the gory of a traumatic accident scene and memory lapse and deterioration of CPR and AED skills) during this time interval may negatively affect the strength of the intention and/or perception of behavioral control. This, in turn, may negatively affect the likelihood of carrying out this behavior. Hence, regular training and re-training as well as refresher courses are of utmost importance. Further works could include expanding the training program on the use of public access AED and bystander CPR training program to other community settings as well.

## Conclusion

In conclusion, this study suggests that a training program is effective in improving the perception and the confidence of the participants towards performing CPR and using AED by training the right people, instilling the right attitude and placing the AEDs in the right locations.

## Data Availability

The dataset is not publicly available due to personal data such as age, gender, occupation, and years of experience; however, it may be requested from the corresponding author upon reasonable request.
